# FgCWM1 modulates TaNDUFA9 to inhibit SA synthesis and reduce FHB resistance in wheat

**DOI:** 10.1186/s12915-024-02007-8

**Published:** 2024-09-11

**Authors:** Yazhou Zhang, Danyu Yao, Xinyu Yu, Xinyao Cheng, Lan Wen, Caihong Liu, Qiang Xu, Mei Deng, Qiantao Jiang, Pengfei Qi, Yuming Wei

**Affiliations:** 1 State Key Laboratory of Crop Gene Exploration and Utilization in Southwest China, and, Triticeae Research Institute, Sichuan Agricultural University, Chengdu, Sichuan 611130 China; 2 National Engineering Laboratory of Crop Molecular Breeding, Institute of Crop Science, Chinese Academy of Agricultural Sciences, Beijing, China; 3 Institute of Phytopathology, Land Use and Nutrition, Research Centre for BioSystems, Justus Liebig University Giessen, Heinrich-Buff-Ring 26, Giessen, 35392 Germany

**Keywords:** Fusarium head blight, Cell wall mannoprotein, Complex I subunit, Interaction, Wheat resistance

## Abstract

**Background:**

Fusarium head blight (FHB) significantly impacts wheat yield and quality. Understanding the intricate interaction mechanisms between *Fusarium graminearum* (the main pathogen of FHB) and wheat is crucial for developing effective strategies to manage and this disease. Our previous studies had shown that the absence of the cell wall mannoprotein *FgCWM1*, located at the outermost layer of the cell wall, led to a decrease in the pathogenicity of *F. graminearum* and induced the accumulation of salicylic acid (SA) in wheat. Hence, we propose that FgCWM1 may play a role in interacting between *F. graminearum* and wheat, as its physical location facilitates interaction effects.

**Results:**

In this study, we have identified that the C-terminal region of NADH dehydrogenase [ubiquinone] 1 alpha subcomplex subunit 9 (NDUFA9) could interact with FgCWM1 through the yeast two-hybrid assay. The interaction was further confirmed through the combination of Co-IP and BiFC analyses. Consistently, the results of subcellular localization indicated that TaNDUFA9 was localized in the cytoplasm adjacent to the cell membrane and chloroplasts. The protein was also detected to be associated with mitochondria and positively regulated complex I activity. The loss-of-function mutant of *TaNDUFA9* exhibited a delay in flowering, decreased seed setting rate, and reduced pollen fertility. However, it exhibited elevated levels of SA and increased resistance to FHB caused by *F. graminearum* infection. Meanwhile, inoculation with the FgCWM1 deletion mutant strain led to increased synthesis of SA in wheat.

**Conclusions:**

These findings suggest that *TaNDUFA9* inhibits SA synthesis and FHB resistance in wheat. FgCWM1 enhances this inhibition by interacting with the C-terminal region of TaNDUFA9, ultimately facilitating *F. graminearum* infection in wheat. This study provides new insights into the interaction mechanism between *F. graminearum* and wheat. *TaNDUFA9* could serve as a target gene for enhancing wheat resistance to FHB.

**Supplementary Information:**

The online version contains supplementary material available at 10.1186/s12915-024-02007-8.

## Background

Wheat (*Triticum aestivum*) is a crucial food crop globally. However, the frequent occurrence of Fusarium head blight (FHB) poses a significant threat to both yield and quality [1]. Wheat grains infected by FHB displayed a deterioration in flour quality and the accumulation of various mycotoxins. Among these mycotoxins, deoxynivalenol inhibits protein synthesis in living organisms, leading to conditions such as anorexia, diarrhea, and vomiting in humans and animals, thereby posing a serious health risk [2]. FHB is a fungal disease caused by a variety of *Fusarium* species, among which *Fusarium graminearum* is one of the most influential and pathogenic strains [3]. The conidia of *F. graminearum* mainly enter through the space between the glumes of wheat. They can penetrate directly into the floret through natural openings or stomata at the flowering stage, colonize, and grow within the tissue cells of wheat [4].

During the infection process, conserved pathogen-associated molecular patterns (such as chitin, chitinase, β-1,3-glucanase, etc.) of *F. graminearum* are identified by pattern recognition receptors on the surface of wheat cells, leading to the accumulation of salicylic acid (SA) in plants [5, 6]. SA could induce the synthesis of pathogenesis-related proteins and reactive oxygen species (ROS) to systemically prepare for resistance [6]. Meanwhile, SA mediated plasmodesmal closure to prevent the hyphae from spreading [7]. A high concentration of SA can also directly inhibit the growth of mycelia [8, 9]. Mitochondrial complex I can supply energy for this process by participating in the conversion of NADH to NAD^+^ [6, 10].

Conversely, *F. graminearum* employs various countermeasures to infect wheat. For example, it can export excess intracellular SA outside the cell or degrade SA by using it as a carbon source to mitigate its toxic effects [8, 9]. In addition, *F. graminearum* releases DON to damage plasma membranes, chloroplasts, and ribosomes, facilitating its colonization of wheat rachis tissues [11]. The release of the orphan secreted protein 24 into wheat cells promoted the binding of ubiquitin-26S proteasome to accelerate the degradation of TaSnRK1α kinase and increased the hyphal spreading in wheat rachis tissues [12]. Although we have made great progress in understanding the interaction between *F. graminearum* and wheat, the molecular mechanisms underlying this complex interaction remain elusive, which limits the genetic improvement of wheat FHB tolerance.

The fungal cell wall plays a crucial role in maintaining cell shape and protecting cells from external damage. It also participates in the early mutual recognition process between the host and pathogen [5, 13, 14]. Our previous studies have shown that FgCWM1 encodes a cell wall mannoprotein (CWM) localized in the outermost part of the cell wall and is highly induced by SA during the process of *F. graminearum* infection [13, 15]. Deletion of FgCWM1 (Δ*Fgcwm1*) resulted in increased sensitivity to SA and decreased pathogenicity and enhanced SA levels in wheat spikelets [16]. CWM is one of the primary components of the outermost layer of the cell wall, and its physical location should be easily recognized by the host plant, leading to interaction effects [17]. Thus, we speculated that FgCWM1 might be involved in the interaction between *F. graminearum* and wheat, affecting SA synthesis in wheat.

In this study, we further searched for proteins that interact with FgCWM1 by screening wheat yeast libraries. The effects of deletion mutants of interacting proteins on disease resistance, SA and ROS were studied. We aimed to analyze the regulatory mechanism underlying the interaction process and identify the weak link in the interaction between *F. graminearum* and wheat. This research provides a new strategy for enhancing wheat resistance to FHB.

## Results

### FgCWM1 interacts with the C-terminal region of TaNDUFA9

**Fig. 1:**
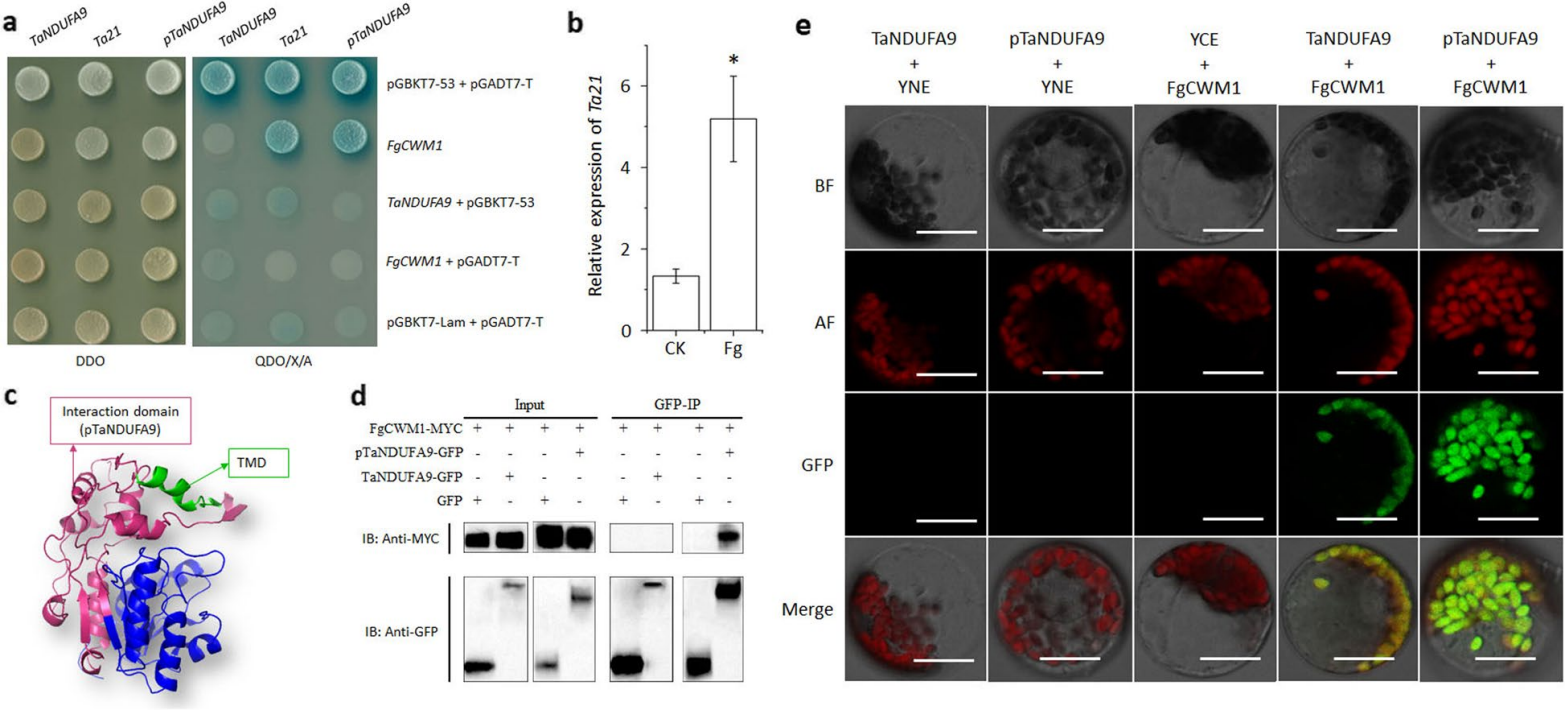
Validation of FgCWM1 interaction with TaNDUFA9 and pTaNDUFA9. **a** Verify the interaction between FgCWM1 and TaNDUFA9 as well as pTaNDUFA9 using the Yeast two-hybrid system. Positive control: pGBKT7-53 + pGADT7-T; negative control: pGBKT7-Lam + pGADT7-T. DDO: SD/-Trp/-Leu. QDO/X/A: SD/-Trp/-Leu/-Ade/-His/X-α-gal/3-AT. **b** Relative expression of TaNDUFA9 in wheat spikes 48 h after *F. graminearum* inoculation. “*” above each box indicates significance at *P* < 0.05 (*n* = 3). **c** Modeling of the tertiary structure of TaNDUFA9 protein. Pink represents the pTaNDUFA9 region, while green represents the transmembrane domain. **d** Verification of the interaction of FgCWM1 with TaNDUFA9 and pTaNDUFA9 by Co-IP. “ + ” represents the addition of the corresponding protein; “-” represents the absence of the corresponding protein. “Anti-MYC” was incubated with the MYC antibody, and “Anti-GFP” was incubated with the GFP antibody. Input refers to total protein, which belongs to the positive control. “IP” refers to the precipitation of the corresponding protein with the corresponding antibody, and “IB” refers to the interaction signal. The original picture is displayed in Additional file 1: Fig. S3. **e** Verification of the interaction of FgCWM1 with TaNDUFA9 and pTaNDUFA9 by BiFC. “BF” represents a bright field of vision, “AF” represents chloroplast autofluorescence, “GFP” represents the green fluorescence signal, and “Merge” represents a composite map with BF, AF, GFP, and RFP. Scale bar = 20 μm

In our previous study, a cell wall mannoglycoprotein, FgCWM1, was shown to be critical for the pathogenicity of *F. graminearum* [16]. To dissect the mechanism of FgCWM1 regulating FHB pathogenicity, we used FgCWM1 as bait to screen for interacting proteins in a wheat cDNA library. Twenty-seven potential interacting proteins were identified through yeast two-hybrid assay (Additional file 2: Table S1), and only *Ta21* was validated to interact with FgCWM1 both in vivo and in vitro (Fig. 1a). The *Ta21* sequences aligned 100% with the 3' UTR and C-terminal sequences of the *TraesCS6A02G389100* by conducting a BLAST search on the EnsemblPlants website (Additional file 1: Fig. S1). Our additional assays revealed that FgCWM1 could interact with the C-terminus of TraesCS6A02G389100.1 but not with the full-length protein in the yeast cells using the yeast two-hybrid method (Fig. 1a). In addition, the expression levels of *Ta21* were dramatically induced under *F. graminearum* inoculation, as quantified by qRT-PCR in the “Kronos” background (Fig. 1b). These results suggest that *TraesCS6A02G389100* may be involved in *F. graminearum* inoculation and have been selected for further study.

TraesCS6A02G389100.1 is predicted to encode a NADH dehydrogenase [ubiquinone] 1 alpha subcomplex subunit 9 (NDUFA9). The phylogenetic analysis showed that TraesCS6A02G389100.1 was most similar to *Arabidopsis* NDUFA9 based on the phylogenetic analysis (Additional file 1: Fig. S2) and was consequently named TaNDUFA9. The predicted tertiary structure of TaNDUFA9 suggests that the interaction region, designated as pTaNDUFA9, is distributed outside the protein and contains a transmembrane domain (321–335 aa) (Fig. 1c).

We further performed a co-immunoprecipitation (Co-IP) assay after the three fusion proteins *TaNDUFA9*-*GFP*, *pTaNDUFA9*-*GFP*, and *FgCWM1*-*MYC* were transiently co-expressed in *Nicotiana tabacum*. A Western blot assay demonstrated that pTaNDUFA9-GFP pulled down FgCWM1-MYC using a GFP-specific resin, confirming the interaction between FgCWM1 and pTaNDUFA9 (Fig. 1d).

A bimolecular fluorescence complementation (BiFC) experiment was also carried out in wheat chloroplasts. The results showed that co-infiltration of *YNE*-*FgCWM1* with *YCE*-*TaNDUFA9* or *YCE*-*pTaNDUFA9* generated a *YFP* fluorescence signal, although the signal of YNE-FgCWM1 co-rotating with YCE-TaNDUFA9 was relatively weaker (Fig. 1e). These results suggest that FgCWM1 strongly interacts with the C-terminus of TaNDUFA9 in *vivo* and in *vitro*.

### TaNDUFA9 is localized around chloroplasts, mitochondria, and the cell membrane

**Fig. 2:**
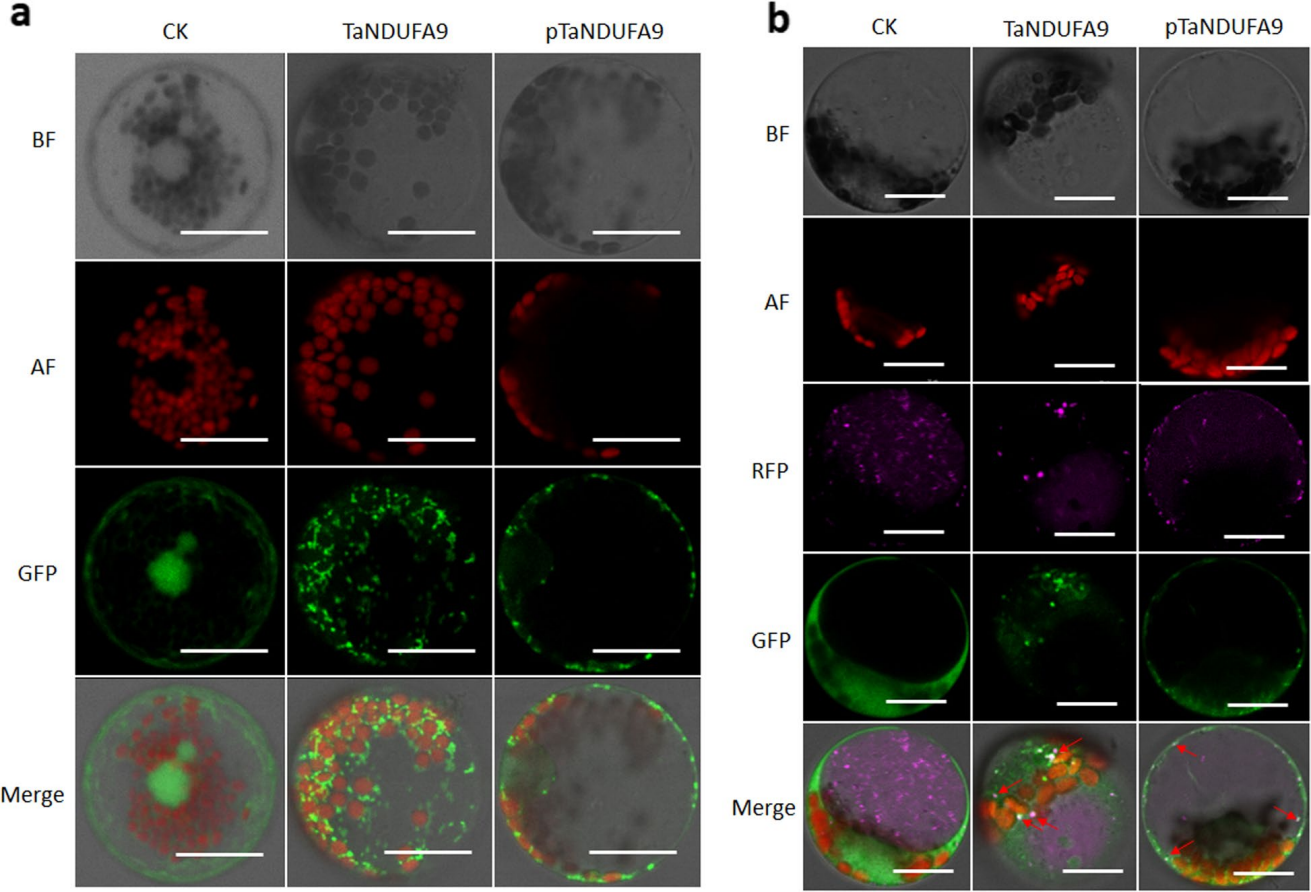
Subcellular localization of TaNDUFA9 and pTaNDUFA9. **a** Subcellular localization of TaNDUFA9 and pTaNDUFA9 in wheat protoplasts. **b** Subcellular localization of TaNDUFA9 and pTaNDUFA9 after the addition of mitochondrial tags. 163-GFP was used as a control. “BF” represents bright field of vision, “AF” represents chloroplast autofluorescence, “GFP” represents a green fluorescence signal, “RFP” represents the red fluorescence signal marked with mitochondrial tags, and “Merge” represents a composite map with BF, AF, GFP, and RFP. Scale bar = 20 μm

TaNDUFA9 was predicted to localize in the cytoplasm and chloroplasts by a website tool (Cell-PLoc 2.0). To verify its subcellular localization, the full-length coding sequence of *TaNDUFA9* was fused to the 5′-terminus of a GFP sequence under the control of a Ubiquitin (UBI) promoter. The *GFP*-fused *TaNDUFA9* and *pTaNDUFA9* were transiently expressed in wheat protoplasts and visualized using scanning confocal microscopy. Imaging revealed that the control group expressing an empty vector exhibited GFP signal throughout the entire cells. In contrast, TaNDUFA9 fused with GFP was predominantly localized in the cytoplasm adjacent to the chloroplast (Fig. 2a). Fluorescence was also found to be associated with mitochondria (Fig. 2b). Similar intracellular distribution was detected in cells expressing GFP fused with pTaNDUFA9 (Fig. 2a, b). These results are consistent with a previous study suggesting that NDUFA9 is a Q-module subunit required for mitochondrial complex I assembly and stabilization [16, 18].

### TaNDUFA9 is involved in complex I activity

**Fig. 3:**
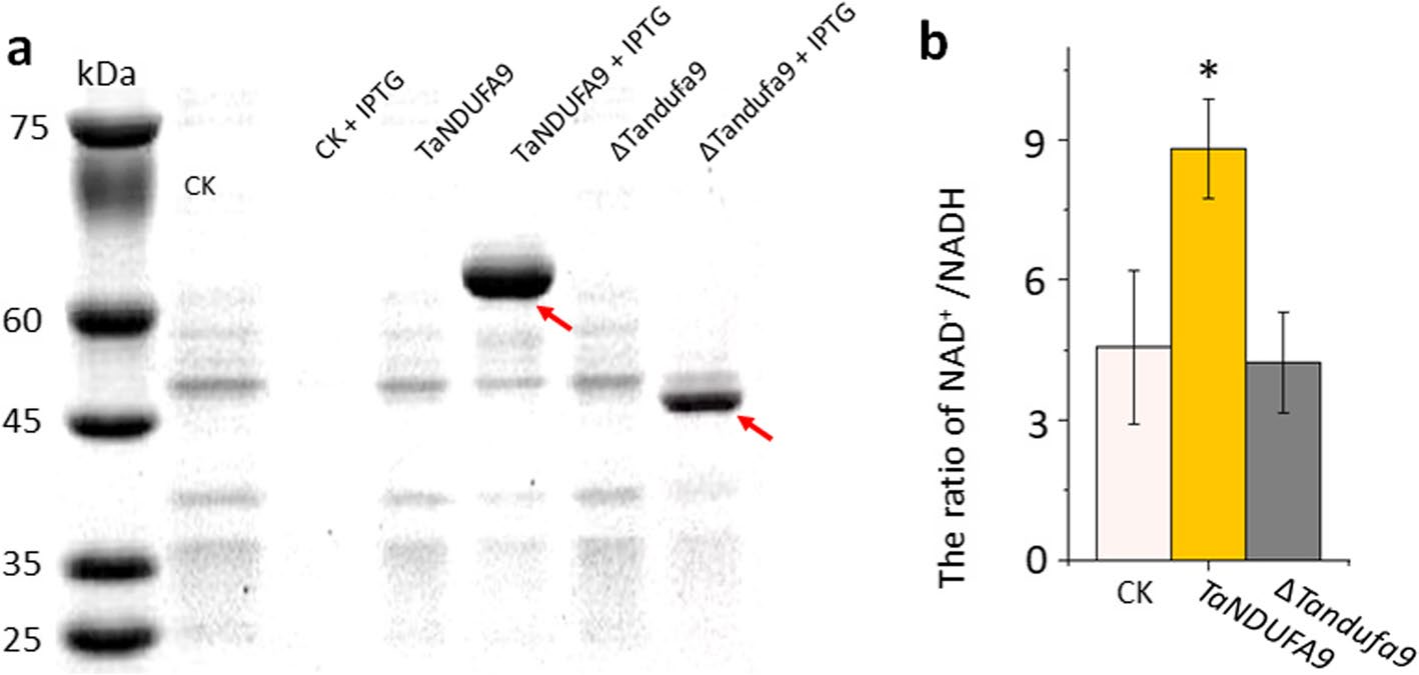
Function analysis of TaNDUFA9 and ∆Tandufa9 in *Escherichia coli.* **a** SDS-PAGE analysis of TaNDUFA9 and ∆Tandufa9 proteins. CK proteins were obtained from *E. coli* cells carrying the pET-32a^(+)^ vector. Red arrows indicate the TaNDUFA9 and ∆Tandufa9 proteins. **b** Detection of NAD^+^/NADH in *E. coli*. pET-32a.^(+)^ was used as the control (CK), while “ + IPTG” indicates treatment with 0.1 mM IPTG. “*” indicates a significant difference, with *P* < 0.05 (*n* ≤ 6, one-way ANOVA for multiple comparisons)

Since NDUFA9 is a Q-module subunit in complex I in mitochondria, we speculate that it participates in the dehydrogenation of NADH to NAD^+^ [16, 19]. To verify this, full-length coding sequences of *TaNDUFA9* and a mutated sequence encoding only the N-terminus of *TaNDUFA9* (designed as ∆*TaNDUFA9*) were inserted into the recombinant pET-32a^(+)^ vector and transformed into *E. coli*. Proteins were purified and analyzed by a sodium dodecyl sulfate–polyacrylamide gel electrophoresis (SDS-PAGE). As expected, TaNDUFA9 displayed a protein size of approximately 60 kDa, while ∆*Tandufa9* was approximately 45 kDa (Fig. 3a). Protein activity in converting NADH to NAD^+^ was measured using a complex I activity kit. The results indicated a significant increase in the NAD^+^/NADH ratio in assays where TaNDUFA9 protein was added compared to the CK (Fig. 3b). These results confirmed that TaNDUFA9, as a part of complex I, promotes the dehydrogenation of NADH into NAD^+^.

### TaNDUFA9 plays an important role in wheat growth

**Fig. 4:**
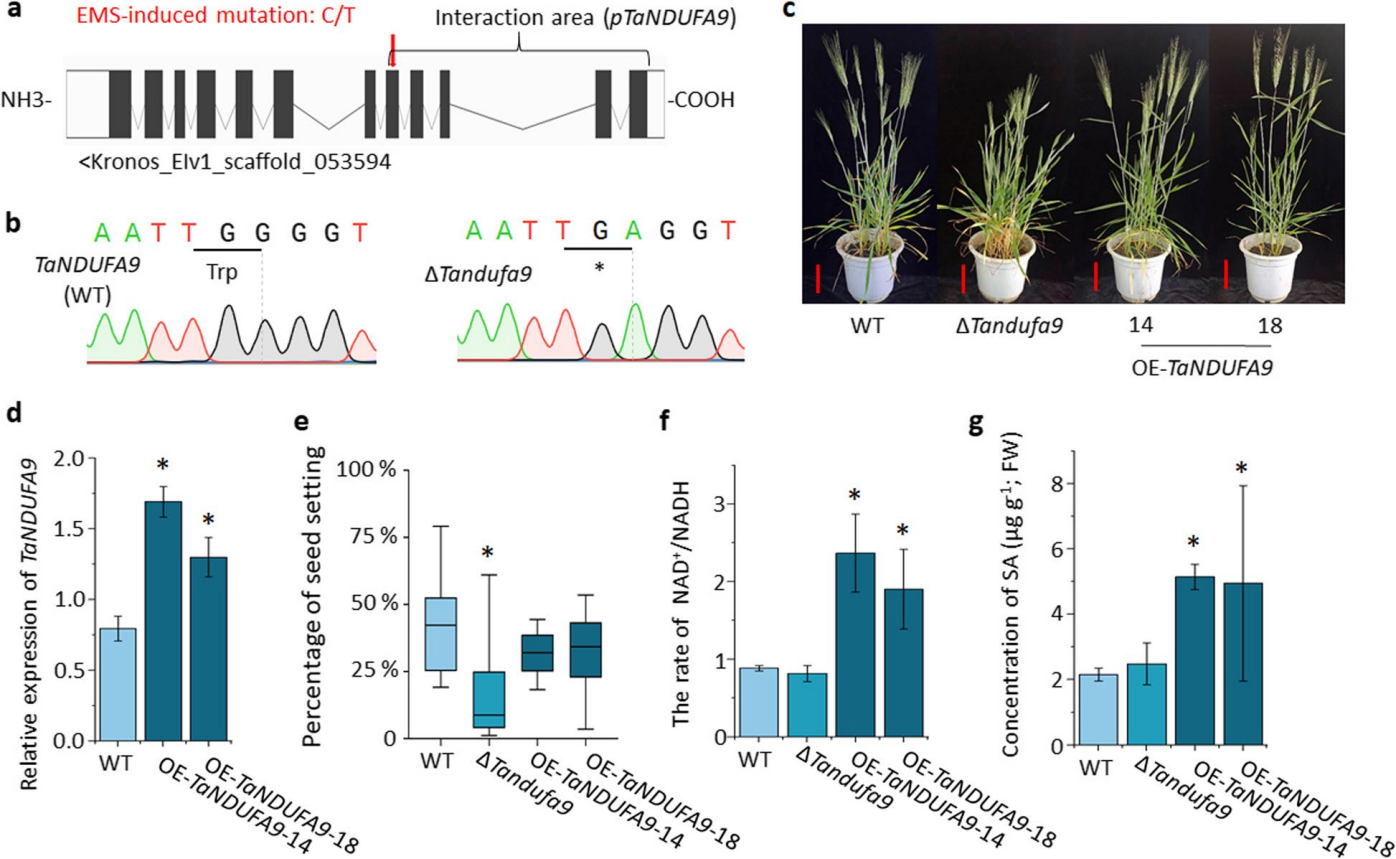
Deletion and complementation of *TaNDUFA9* in wheat. **a** Schematic diagram of *TaNDUFA9* mutation site. **b** Verification of ∆*Tandufa9* mutation location by sequencing. **c** Phenotype of WT, ∆*Tandufa9*, OE-*TaNDUFA9*-14, and OE-*TaNDUFA9*-18 at Zadok60 stage; scale bar = 10 cm. **d** Relative quantitative results of *TaNDUFA9* in transgenic plants leaves at Zadok Scale 29 (*n* = 3). **e** Percentage of seed setting in mutant and transgenic lines, with WT as control (*n* ≥ 40). **f** Determination of complex I activity by detecting the rate of NAD.^+^/NADH in mutant and transgenic lines spikelets at Zadok60 stage, with WT as control (*n* = 3). **g** SA content was measured in mutant and transgenic lines spikelets at the Zadok 60 stage, with WT used as the control (*n* = 3). One-way ANOVA for multiple comparisons. “*” indicates a significant difference, with *P* < 0.05

To further investigate the functional role of *TaNDUFA9* in wheat growth and development, we screened the nonsense mutant from the ethyl methanesulfonate (EMS)–mutagenized Kronos library and identified one mutant, aaBB (T4-4520), of *TaNDUFA9*. The mutation was confirmed by Sanger sequencing, which leads to an amino acid change to a stop codon in the C-terminal region of the protein (Fig. 4a, b). The cDNA sequences of mutated *TaNDUFA9* were inserted into the recombinant pET-32a^(+)^ vector for SDS-PAGE and complex I activity testing. The results indicated that nonsense mutations in *TaNUDFA9* resulted in the loss of activity in complex I (Fig. 3b). We backcrossed the mutant twice with wild type (WT) to obtain BC_2_ lines. Phenotypic analysis showed that ∆*Tandufa9* displayed stunted growth, such as delayed flowering, decreased seed setting rate, and reduced pollen fertility (Fig. 4c, Additional file 1: Fig. S4).

Meanwhile, we created *TaNDUFA9* overexpressing construct driven by a UBI promoter and transformed it into ∆*Tandufa9*. Ten positive transgenic lines were obtained, and lines OE-*TaNDUFA9*-14 and OE-*TaNDUFA9*-18, which exhibited a higher transcript level of *TaNDUFA9* than WT plants, were selected for further analysis (Fig. 4d). Transgenic plants overexpressing *TaNDUFA9* only partially complemented the growth retardation of mutant plants but showed increased activity of complex I (Fig. 4c, e, f). This was consistent with the data of in vitro assays (Fig. 3b). Together, these results indicate that *TaNDUFA9* is involved in complex I activity and SA accumulation and is indispensable for normal growth and development in wheat.

### TaNDUFA9 negatively regulates wheat FHB resistance

**Fig. 5:**
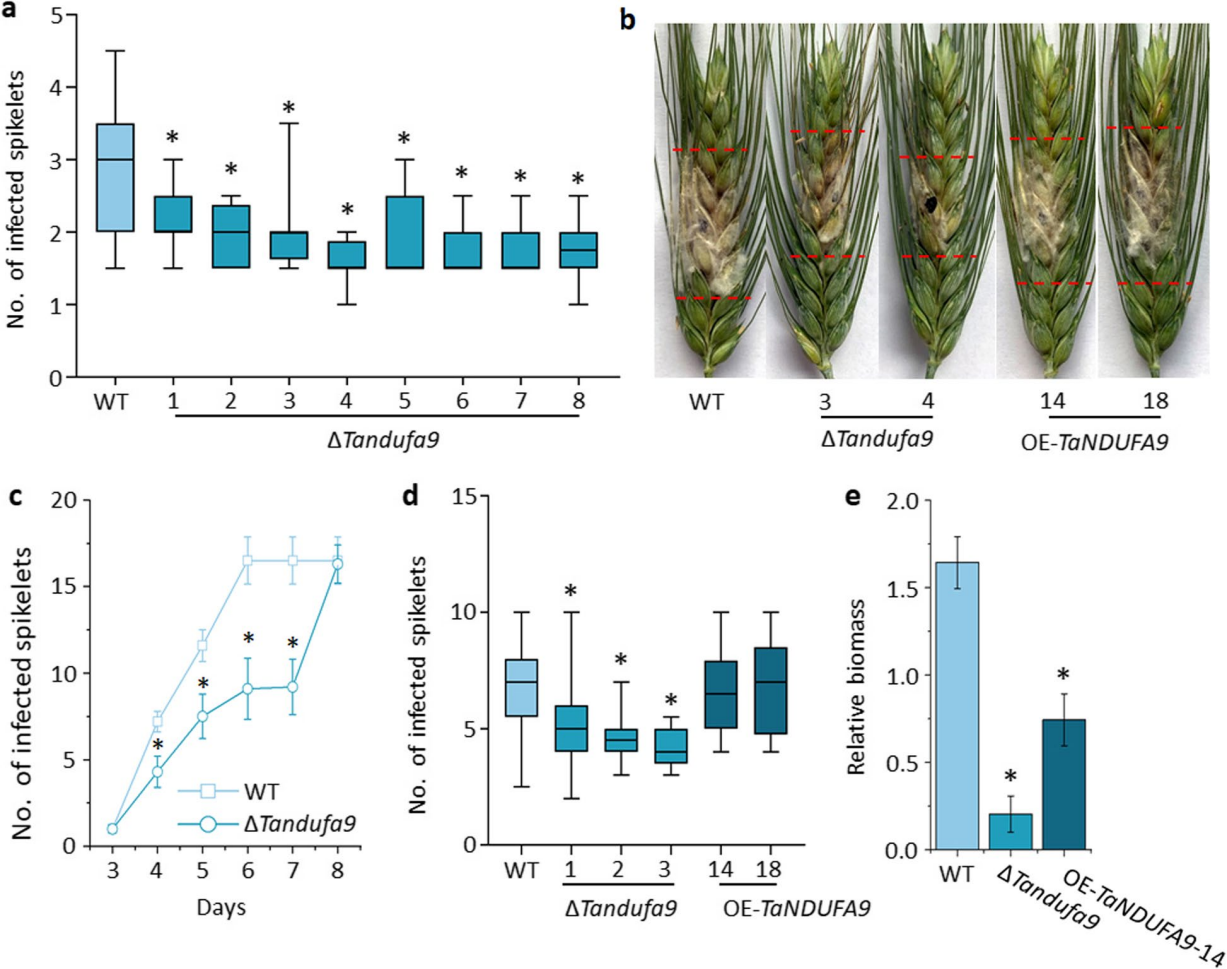
Effects of *TaNDUFA9* on FHB resistance. **a** The number of infected spikelets was recorded 17 days after inoculation with Fg in field condition. Eight homozygous mutants were selected for the FHB resistance test (*n* = 20). **b** Phenotype of WT and ∆*Tandufa9* on the 5 days after inoculation with 1 × 10.^3^ conidia in vitro. **c** The number of infected spikelets was recorded 5 days after inoculation with Fg in vitro, three homozygous mutants were selected for the FHB resistance test (*n* = 10). **d** The number of infected spikelets was recorded 10 days after inoculation with Fg in greenhouse condition. Three homozygous mutants were selected for the FHB resistance test (*n* = 20). **e** Relative fungal biomass in wheat spikes 48 h after *F. graminearum* inoculation (*n* ≥ 6). One-way ANOVA for multiple comparisons. “*” indicates a significant difference, with *P* < 0.05

To investigate the influence of TaNDUFA9 on FHB resistance, we inoculated *F. graminearum* to WT and Δ*Tandufa9* plants in the field. By analyzing the infected spikelets, we found that Δ*Tandufa9* was more resistant to FHB compared with WT plants (Fig. 5a). These results were further confirmed by inoculated *F. graminearum* to WT, Δ*Tandufa9*, and OE-*TaNDUFA9* plants in the greenhouse (Fig. 5b). Relative biomass results also showed that Δ*Tandufa9* had significantly reduced total fungal biomass when compared to WT (Fig. 5c). Consistently, our results indicated that *TaNDUFA9* plays a negative role in wheat resistance to *F. graminearum* infection.

### The interaction between FgCWM1 and TaNDUFA9 affects the synthesis of SA in wheat

**Fig. 6:**
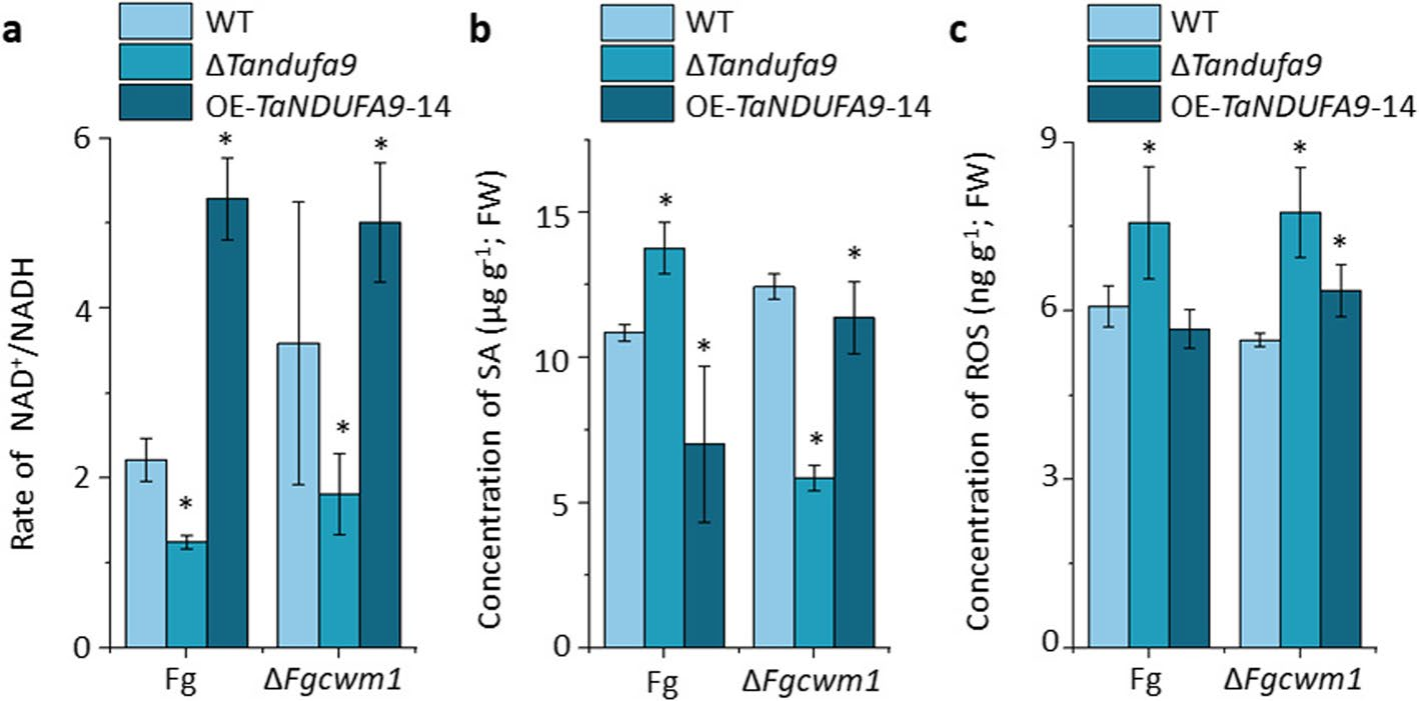
The impact of *TaNDUFA9* on NAD^+^/NADH, SA, and ROS accumulation. **a** Determination of complex I activity by detecting the rate of NAD.^+^/NADH in WT, ∆*Tandufa9*, and OE-*TaNDUFA9*-14 spikelets at 48 h after inoculation with Fg and ∆*Fgcwm1* (*n* = 3). **b** SA content in WT, ∆*Tandufa9*, and OE-*TaNDUFA9*-14 spikelets at 48 h after inoculation with Fg and ∆*Fgcwm1* (*n* = 3). **c** ROS content in wheat spikelets at 48 h after inoculation with Fg and ∆*Fgcwm1* (*n* = 3). One-way ANOVA for multiple comparisons. “*” indicates a significant difference, with *P* < 0.05

To investigate the impact of the interaction between FgCWM1 and TaNDUFA9 on the changes in complex I activity and SA concentration in wheat spikelets, Fg and ∆*Fgcwm1* were employed to inoculate the WT, ∆*Tandufa9*, and OE-*TaNDUFA9* plant lines at Zadok stage 65, respectively. Consistent with previous results, the NAD^+^/NADH concentration was obviously higher in *TaNDUFA9* overexpressing lines than that in WT and mutant plants following the inoculation of both Fg strains. In addition, the NAD^+^/NADH ratio in WT plants increased when infected with ∆*Fgcwm1* instead of Fg, suggesting that the interaction of TaNDUFA9 and FgCWM1 may affect the NAD^+^/NADH balance (Fig. 6a). In contrast, SA contents in all plant lines were reverse correlated with the NAD^+^/NADH concentration following the inoculation of Fg. SA levels enhanced in WT and *TaNDUFA9* overexpressing lines when plants were inoculated with a mutated strain lacking the FgCWM1 protein (Fig. 6a, b). These results indicate that *TaNDUFA9* negatively affects SA synthesis, and the interaction of FgCWM1 with TaNDUFA9 can promote the inhibitory effect.

The results of ROS content determination showed that the loss of *TaNDUFA9* significantly increased ROS accumulation in spikelets after infection with either Fg or ∆*Fgcwm1* strain (Fig. 6c), indicating that TaNDUFA9 is an inhibitory protein for ROS synthesis in wheat independent of FgCWM1.

## Discussion

**Fig. 7:**
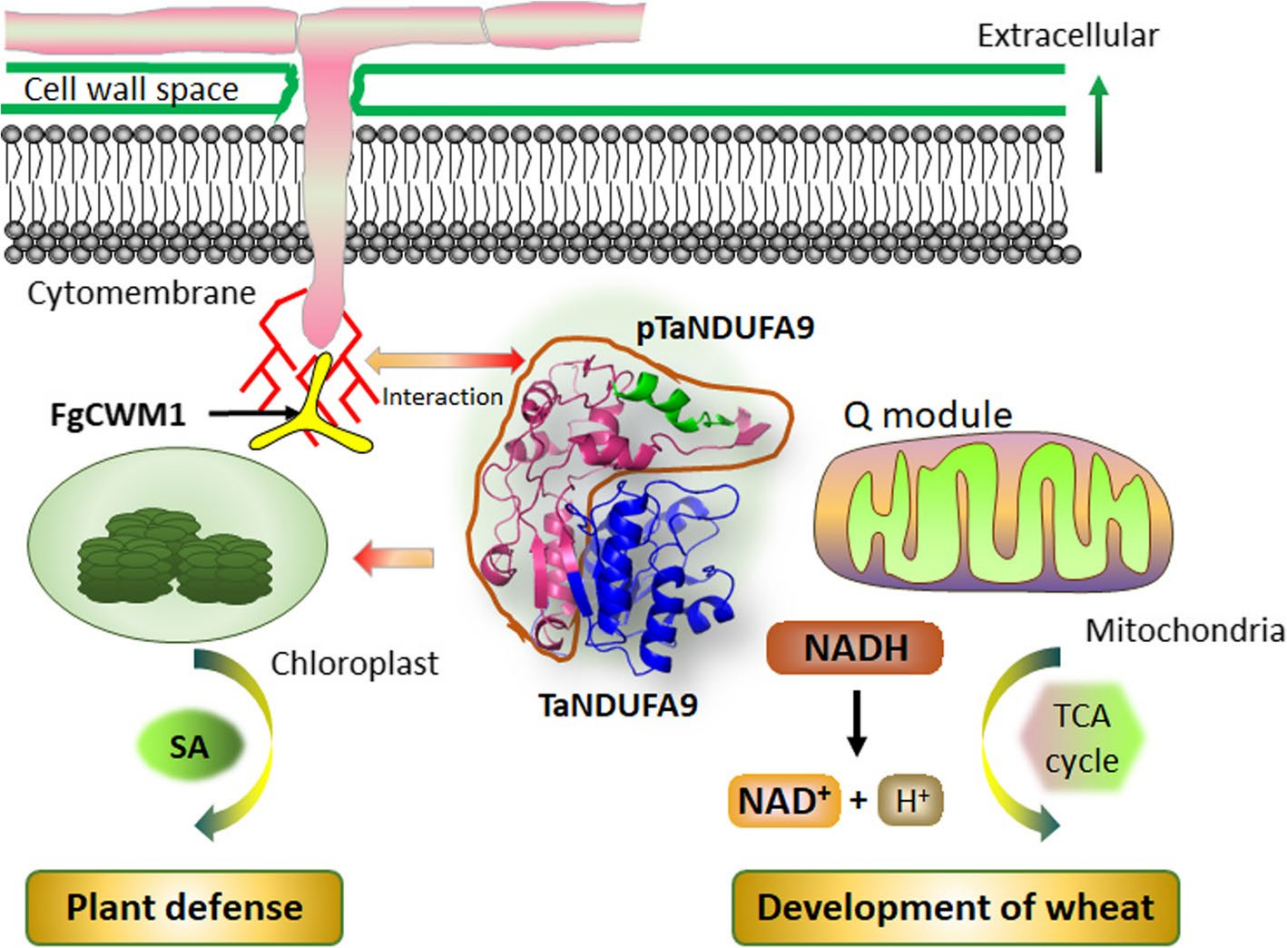
Model of the interaction mechanism between FgCWM1 and TaNDUFA9

Fungal cell walls are composed of polysaccharides and proteins, serving a crucial role in facilitating fungal colonization, host recognition, and the initiation of disease resistance [17]. Chitin plays an important role in eliciting plant immune responses. However, chitin deacetylase converts chitin to chitosan to evade chitinase degradation and escape detection by chitin receptors in the plant immune system [20, 21]. Additionally, the antifungal protein AFP1 interacts with various chitin deacetylases by binding to the conserved NodB domain, which disrupts their enzyme activity [22]. Mannans are a significant virulence factor associated with the severity and pathogenesis of Candida infections and can be recognized by Toll-like receptors in the host [23]. Indeed, despite significant advancements in the understanding of fungal cell biology, the complexity of fungal cell walls presents ongoing challenges, and there remain numerous unanswered questions in this field. The CWM is situated in the outermost layer of the cell wall of *F. graminearum*. It is unexpected that the interaction and recognition reactions take place when wheat fungi infect wheat due to the presence of this crucial component [16, 17]. In this study, we found that FgCWM1 interacts with the C-terminal region of wheat TaNDUFA9, influencing the accumulation of SA and ROS, consequently diminishing wheat's resistance to FHB (Fig. 7). This study offers new insights into the molecular basis of plant-fungus interactions.

In plants, the biology of complex I is particularly intriguing as it is involved in the dehydrogenation of NADH to NAD^+^. Recently, studies have demonstrated that it plays a crucial role in oxidoreductase activity, proton translocation, respiration, photorespiration, photosynthesis, carbonic anhydrase function, and other processes [10, 19]. NDUFA9 is a Q-module subunit required for the assembly and stabilization of mitochondrial complex I, which contains more than 40 subunits in plants [10, 18]. The deletion of various complex I subunits can lead to different physiological defects in plants. For instance, the loss of function of *NAD7*, *NDUFS4*, or *NDUFV1* can lead to delayed growth and development in *Nicotiana tabacum* and *Arabidopsis thaliana* [24, 25]. The OsNDUFA9 subunit of complex I was localized in the mitochondria and is crucial for embryo development and starch synthesis in rice endosperm [26]. In this study, we found that TaNDUFA9 was distributed around mitochondria, chloroplasts, and cell membranes and involved in the activity of complex I in wheat (Figs. 2, 3b, and 4d). Consistent with previous studies, our phenotypic analyses showed that ∆*Tandufa9* plants exhibit weakened growth, decreased seed setting rates, and reduced pollen fertility in wheat (Fig. 4c, e, Additional file 1: Fig. S4). In addition, we also found that overexpression of *TaNDUFA9* induced the synthesis of NAD^+^ and SA in wheat spikelets (Fig. 4f, g). Qiao et al. [27] also found that *PuNDH9*, as a subunit of complex I, played key roles in NAD^+^/NADH homeostasis, ROS generation, and mitochondrial functions during the pear-*A. alternata* interaction and controls a defense pathway mediated by SA. Since plant chloroplasts are the primary organelles for synthesizing [6], TaNDUFA9 and FgCWM1 will be relocated to chloroplasts following co-transformation (Fig. 1e). We suggest that *TaNDUFA9* not only affects the growth and development of plants by influencing the balance of NAD^+^ and NADH but also plays a crucial role in SA synthesis in wheat chloroplasts under *F. graminearum* infection (Fig. 7).

In this study, although the ratio of NAD^+^/NADH in ∆*Tandufa9* was significantly lower than that in WT and OE-*TaNDUFA9* under *F. graminearum* infection, the concentration of SA and ROS was significantly higher than that of WT and OE-*TaNDUFA9*, resulting in enhanced resistance to FHB. This result is consistent with the findings demonstrating decrease in NAD^+^ content and induction of SA synthesis in *Arabidopsis thaliana* due to the absence of *AtNUDX19* [28]. This may be due to the fact that other diverse complex I subunits in wheat may help maintain the balance of NADH and NAD^+^ and promote the accumulation of SA [10]. The absence of FgCWM1-TaNDUFA9 interaction resulted in the elevated SA and ROS concentration but decreased NAD^+^/NADH ratio in plants, ultimately promoting wheat resistance to FHB.

FHB poses a significant threat to wheat production and has serious implications for food security. With the emergence of resistance to commonly used antifungals, it is crucial to intensify the search for new targets to effectively combat this disease [29]. NDUFA9 features a single domain that includes a structurally conserved Rossmann fold, an NAD(P)(H)-binding region, and a structurally diverse C-terminal region [30]. The C-terminal of *TaNDUFA9* exhibits numerous variants in 1769 hexaploid wheat (Additional file 1: Fig. S5). These variants may hinder the interaction with FgCWM1, enhancing wheat resistance to FHB, thereby avoiding undesirable traits such as delayed flowering and reduced seed setting rate.

## Conclusions

By assays in vitro and in vivo, the C-terminal region of TaNDUFA9 was verified to interact with FgCWM1. TaNDUFA9 was shown to participate in complex I activity and essential for normal plant growth. In addition, TaNDUFA9 negatively modulated wheat FHB resistance. The interaction between FgCWM1 and TaNDUFA9 appears to enhance the inhibitory effect on disease resistance. This study provides new insights into the interaction mechanism between *F. graminearum* and wheat. *TaNDUFA9* could be considered as a target gene for enhancing wheat resistance to FHB.

## Methods

### Plant materials and growth conditions

All the mutants and transgenic wheat used in this study were in *Durum wheat* (*Triticum turgidum* L. ssp. *Durum* (Desf.) Husn.) (AABB genome) “Kronos” background, with middle susceptible to FHB. Early termination mutants (T4-4520, ∆*Tandufa9*, aaBB) were obtained from EMS mutagenized population of “Kronos.” All the mutants were backcrossed each of the selected mutants with the non-mutagenized Kronos twice to reduce background influence. Based on Derived Cleaved Amplified Polymorphic Sequences (dCAPS) markers (Additional file 2: Table S2), all two possible homozygous BC_2_F_2_ (WT and ∆*Tandufa9*) were selected. Two homozygous combinations were further selected at BC_2_F_8_, and ten lines for each homozygous combination were selected for phenotypic analyses. All transgenic plants were generated following the procedures outlined in a previous study [31]. The T_1_ lines obtained were verified by primer pair Ubi1899F + Bar496R (Additional file 2: Table S2). Phenotypic analyses were completed with the T_2_ generation, and the results were confirmed with the T_3_ generation. All lines were cultivated in greenhouses under 16/8-h day/night cycles at 25/16 °C, watered as necessary, and fertilized with 15–15-15 (N-P-K) before sowing. All mutants and Chinese Spring nullisomic-tetrasomic lines were stored at the Triticeae Research Institute, Sichuan Agricultural University.

### Sequence analysis and primer design

The nucleotide sequence of TaNDUFA9 was downloaded from the Jorge Dubcovsky Lab database (https://dubcovskylab.ucdavis.edu/). The CDS sequence was predicted based on the *Triticum turgidum* Svevo.v1 database, which has been published on Ensembl Plants (http://plants.ensembl.org/index.html), and confirmed by sequencing. Primer Premier 5.0 software (Premier Biosoft, Palo Alto, Canada) was utilized to design PCR primers (Additional file 2: Table S2). Neighbor-joining trees (1000 replicates) for the classification of deduced proteins were constructed using the MEGA software. The trees were based on Poisson correction and complete deletion of gaps. DNAman 9.0 (LynnonBiosoft, San Ramon, CA, USA) was used to perform multiple sequence alignments.

### Protein interaction assays

Total RNAs were extracted from wheat spikelets (cultivar: sumai482, middle susceptible to FHB) without inoculation of *F. graminearum* using Trizol reagent (Invitrogen, CA, USA). After pooling equal quantities of RNAs from each treatment, the pooled mRNA was purified with a FastTrack MAG mRNA Isolation Kit (Invitrogen, CA, USA). The SuperScript III First-Strand Synthesis System (Invitrogen, CA, USA) was used for reverse transcription. A wheat cDNA library was prepared using a CloneMiner II cDNA Library Construction Kit (Invitrogen, CA, USA) following the manufacturer’s instructions. For screening interaction proteins of *FgCWM1*-BD, the bait plasmid *FgCWM1*-BD and pGADT7-cDNA libraries were co-transformed into cells of the yeast strain AH109 using the Yeastmaker™ Yeast Transformation System 2 (Clontech, CA, USA) following the manufacturer’s instructions. Positive yeast clones were then selected on the SD/–Trp-Leu-His medium. Clones from SD/–Trp-Leu-His were randomly selected for culture on the SD/–Trp/–Leu/–His/–Ade medium. Subsequently, we selected the positive clones for sequencing to determine the candidate targets. The candidate target sequences were further cloned from wheat and constructed into the prey vector pGADT7. *FgCWM1*-BD co-transforms into the yeast strain AH109 with all candidate target sequences separately, and their interaction was confirmed by plating on the medium lacking Leu-Trp-His-Ade.

For the Co-IP assay in *N. benthamiana*, Agrobacteria carrying recombinant plasmids were adjusted to a concentration of OD_600_ = 0.6–1.0 and infiltrated into 5-week-old *N. benthamiana* leaves. Total protein extracts were isolated for Co-IP experiments at 2 days post infiltration and detected by Western blotting using an anti-MYC antibody (ABclonal, MA, USA) and an anti-GFP antibody (Roche, NY, USA).

For the BiFC assay in wheat protoplasts, the coding regions of *FgCWM1*, *TaNDUFA9*, and *pTaNDUFA9* were inserted into pUC-SPYCE or pUC-SPYNE, respectively. Prepare protoplasts according to earlier reports [31]. The recombinant constructs were co-expressed in “Kronos” protoplasts and then observed using confocal laser scanning microscopy (Leica EM CPD300, IL, USA).

### Subcellular localization

For subcellular localization analysis, the coding sequence of *TaNDUFA9* was incorporated into 163-*hGFP* (Pro UBI: *TaNDUFA9*-*hGFP*) by LR reaction (Invitrogen, CA, USA) and then transformed into protoplasts of “Kronos” following previous reports [31]. The localization of TaNDUFA9 in wheat protoplasts was observed using a Leica microscope (Leica Microsystems Trading Co, Shanghai, China).

### Determination of complex I activity

To prepare *Escherichia coli* samples, the coding regions of *TaNDUFA9* and ∆*TaNDUFA9* were incorporated into pET-32a^(+)^ and transformed into *E. coli* BL21 (DE3) cells (Tiangen, Beijing, China). All *E. coli* cells were cultured in Luria–Bertani liquid medium until reaching an optical density of 0.6 at 600 nm (OD_600_). The production of TaNDUFA9 and ∆TaNDUFA9 was induced by the addition of 0.1 mM isopropylβ-D-1-thiogalactoside and incubation with shaking (180 rpm) for 4 h at 37 °C. The *E. coli* cells carrying pET-32a^(+)^, pET-32a^(+)^-*TaNDUFA9*, and pET-32a^(+)^-∆*TaNDUFA9* were collected for NAD^+^ and NADH testing.

To prepare wheat spike samples, two florets from each fully developed spikelet in a whole spike at the mid-anthesis stage were inoculated with 1 × 10^3^ conidia, and the humidity was increased to 80%. The inoculated wheat plants were treated as described above. Forty-eight-hour-inoculated spikes that have a significant difference in SA content were harvested and ground to a fine powder in liquid nitrogen [16].

The levels of NAD^+^ and NADH were determined using the Activity Kit for Complex I (Solarbio, Beijing, China), following the manufacturer’s instructions. To rule out differences in the number of cells in the sample, the ratio of NAD^+^ to NADH (NAD^+^/NADH) was used for the final data analysis. Three biological replicates were used per treatment.

### FHB resistance assay

Based on Zadoks’ cereal development scale, the Zadoks 60 stage of wheat spikelets was used in FHB resistance assay. All experiments were conducted with the *F. graminearum* isolate DAOM180378 (Fg, Canadian Fungal Culture Collection, AAFC, Ottawa, ON, Canada), which is highly virulent in wheat. Two flowering florets of a central spikelet of one head were each inoculated with 1 × 10^3^ conidia. The inoculated heads were sprayed with water and enclosed with plastic wrap for 48 h at 25 °C. Wheat plants were placed in a controlled environment room at 25 °C. FHB symptoms were assessed 10 days after inoculation. Twenty plants were used per line. All lines were cultivated in greenhouses under 16/8-h day/night cycles at temperatures of 25/16 °C, with humidity maintained at ≥ 60%. For FHB resistance assay in vitro, the spikes inoculated as described above were cut off from the plant and placed in an environment with humidity controlled at ≥ 85%. Ten lines of each homozygous mutant and transgenic lines were selected for FHB resistance assay, and ten spikes were used in each line. Fungal biomass was estimated using the expressed levels of *FgGAPDH* and determined according to previous study [13].

### Gene expression analysis

Total RNA was extracted from fresh wheat spikelet powders using the E.Z.N.A.® Total RNA Kit I (Omega Bio-Tek, Norcross, GA, USA) following the manufacturer’s instructions. RNA was reverse transcribed using the PrimeScript™ RT Reagent Kit with genomic DNA Eraser (Takara, Dalian, China) following the manufacturer’s protocol. The *TaGAPDH* (*Ta.66461*) and *TaAox* (*Ta.6172*) genes were used as references when performing qPCR for the samples of *Triticum turgidum* (Additional file 2: Table S2). qPCR was performed using a MyiQ Real-Time PCR Detection System (Bio-Rad, CA, USA).

### Quantification of SA and ROS in wheat spikes

The preparation methods for measuring SA and ROS content in wheat spikelets were consistent with those for measuring NAD^+^/NADH. The content of SA was determined by HPLC according to earlier reports [32]. The chromatographic results were analyzed using an Agilent (CA, USA) C_18_ Eclipse (5 μm, 2.1 × 150 mm) and the OpenLab CDS (Agilent Technologies, CA, USA) software. The content of ROS was determined using the Plant ROS ELISA Kit (Lincheng Biological Technology, Beijing, China), according to the manufacturer’s instructions. Analyses were completed with three biological replicates per treatment.

### Statistical analysis

The GraphPad Prism 8 software (GraphPad, MA, USA) was utilized to assess the significance of differences among the average values of infected spikelets number, SA content, and gene expression. Statistical differences were analyzed using the least significant difference test at *P* ≤ 0.05. In order to minimize errors, two independent tests were performed, and the average value of the two tests was taken as the final result.

## Supplementary Information

Additional file 1: Figures S1-S5. Figure S1. Sequence alignment of *TaNDUFA9*, *Ta21*, and *pTaNDUFA9*. Figure S2. Phylogenetic analysis of TaNDUFA9 protein sequences in wheat by Neighbor-joining tree. Figure S3. The original image of the CO-IP experiment confirms the interaction between FgCWM1 and TaNDUFA9, as well as pTaNDUFA9. Figure S4. Pollen fertility of WT, ∆*Tandufa9*, and OE-TaNDUFA9 was assessed using 1% (v/v) Lugol’s solution staining. Figure S5. Schematic of *TaNDUFA9* variation in wheat union database [33].

Additional file 2: Table S1-S2. Table S1. Potential interaction sequences were obtained by screening wheat yeast libraries. Table S2. Primers used in this study.

Additional file 3: Supporting data values file.

## Data Availability

All data generated or analyzed during this study are included in this published article, its supplementary information files, and publicly available repositories. Supporting data values for *n* < 6 individual data values reported in the figures are detailed in the Additional file 3: Supporting data values file.

## Abbreviations

FHB: Fusarium head blight
SA: Salicylic acid
NDUFA9: NADH dehydrogenase [ubiquinone] 1 alpha subcomplex subunit 9
ROS: Reactive oxygen species
CWM: Cell wall mannoprotein
UBI: Ubiquitin
SDS-PAGE: Sodium dodecyl sulfate–polyacrylamide gel electrophoresis
CK: Control
EMS: Ethyl methanesulfonate
WT: Wild type
dCAPS: Derived Cleaved Amplified Polymorphic Sequences
BiFC: Bimolecular fluorescence complementation
Co-IP: Co-immunoprecipitation

## Data availability

No datasets were generated or analysed during the current study.
