# Supplementary material for: FgCWM1 modulates TaNDUFA9 to inhibit SA synthesis and reduce FHB resistance in wheat
Source: BMC Biol. 2024 Sep 11;22:204. doi: 10.1186/s12915-024-02007-8 (PMC11389325; doi:10.1186/s12915-024-02007-8)
Supplement: Supplementary file 1 — Additional file 1: Figures S1-S5. Figure S1. Sequence alignment of TaNDUFA9, Ta21, and pTaNDUFA9. Figure S2. Phylogenetic analysis of TaNDUFA9 protein sequences in wheat by Neighbor-joining tree. Figure S3. The original image of the CO-IP experiment confirms the interaction between FgCWM1 and TaNDUFA9, as well as pTaNDUFA9. Figure S4. Pollen fertility of WT, ∆Tandufa9, and OE-TaNDUFA9 was assessed using 1% (v/v) Lugol’s solution staining. Figure S5. Schematic of TaNDUFA9 variation in wheat union database [33]. [file 12915_2024_2007_MOESM1_ESM.docx]

**
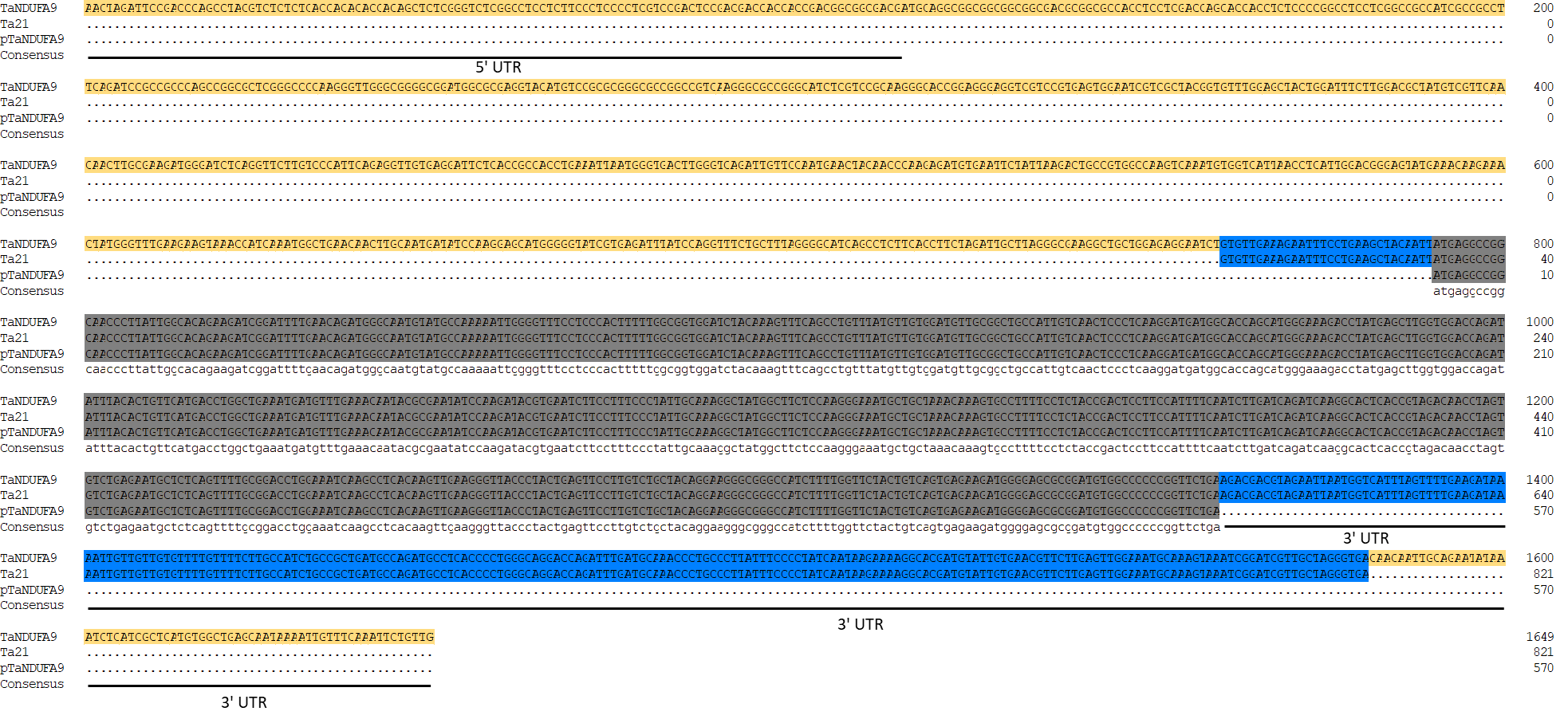
**

**Figure S1.** Sequence alignment of *TaNDUFA9*, *Ta21*, and *pTaNDUFA9*.


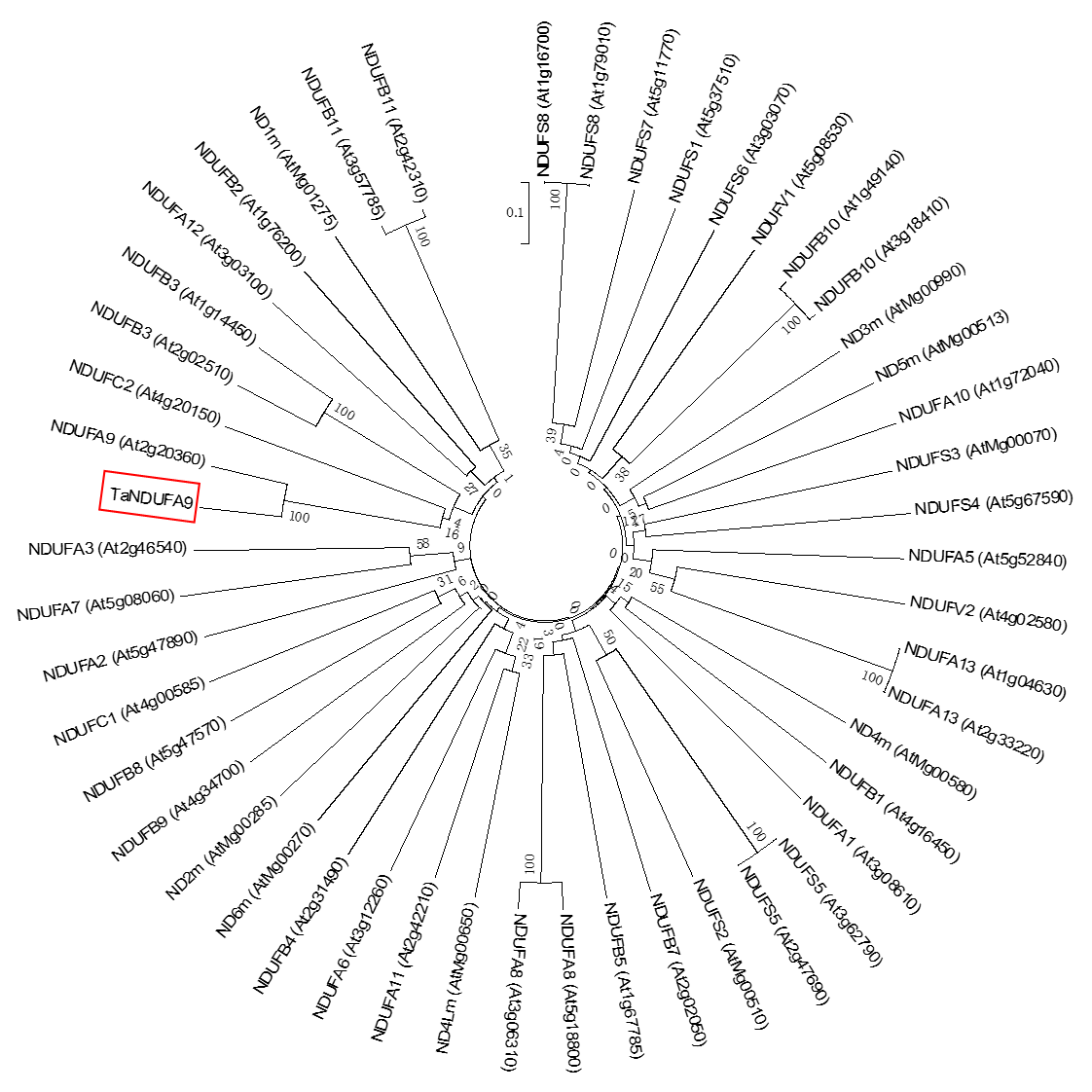


**Figure S2.** Phylogenetic analysis of TaNDUFA9 protein sequences in wheat by Neighbor-joining tree. Bootstrap test of phylogeny was conducted with 10,000 replicates, and branch length values were displayed at the nodes of each proposed class. All detailed information regarding the Arabidopsis protein sequence was referenced from a previous study [10].

**
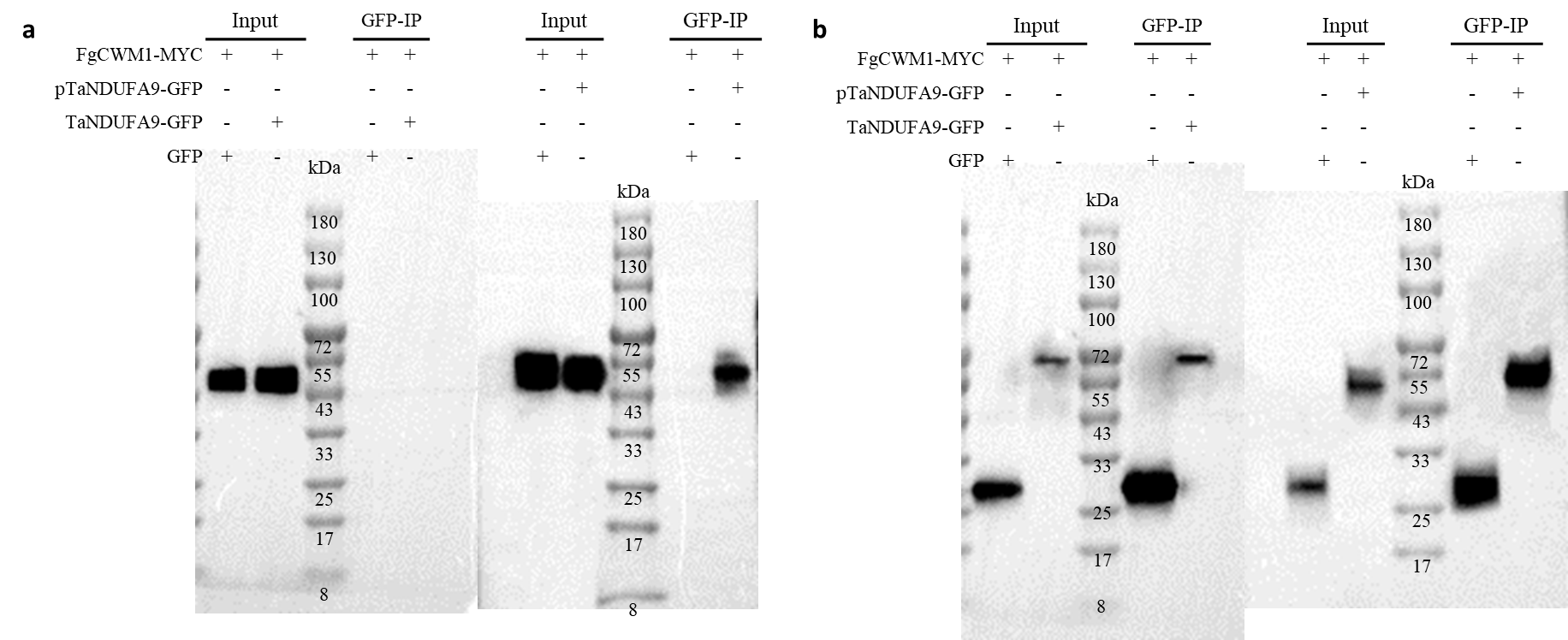
**

**Figure S3. The original image of the CO-IP experiment confirms the interaction between FgCWM1 and TaNDUFA9, as well as pTaNDUFA9. 'a' was incubated with the MYC antibody, while 'b' was incubated with the GFP antibody.**

**
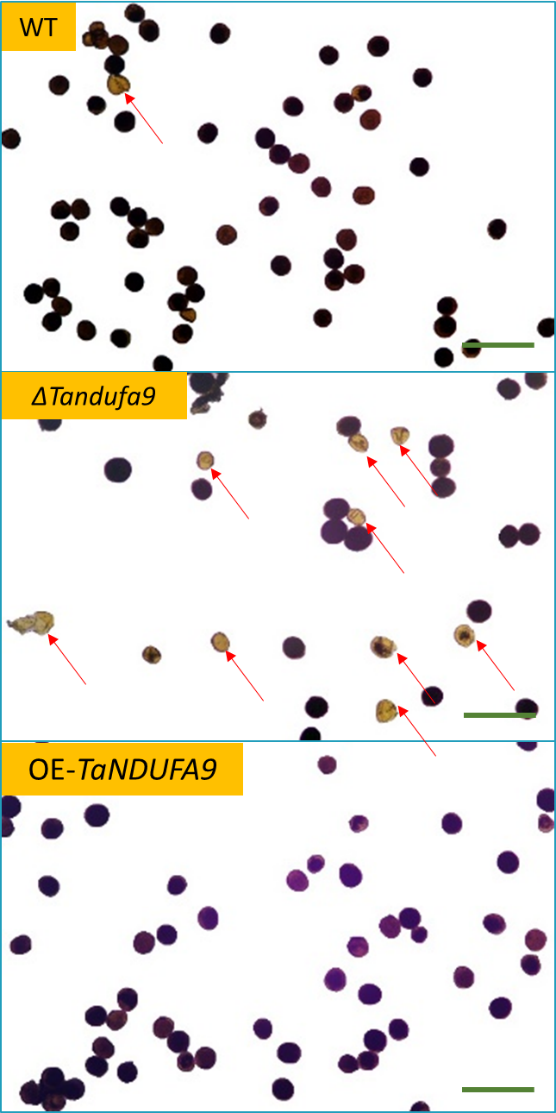
**

**Figure S4.** Pollen fertility of WT, ∆*Tandufa9*, and OE-TaNDUFA9 was assessed using 1% (v/v) Lugol's solution staining. The sterile pollen was marked with a red arrow. Scale bar = 40 μm.

**
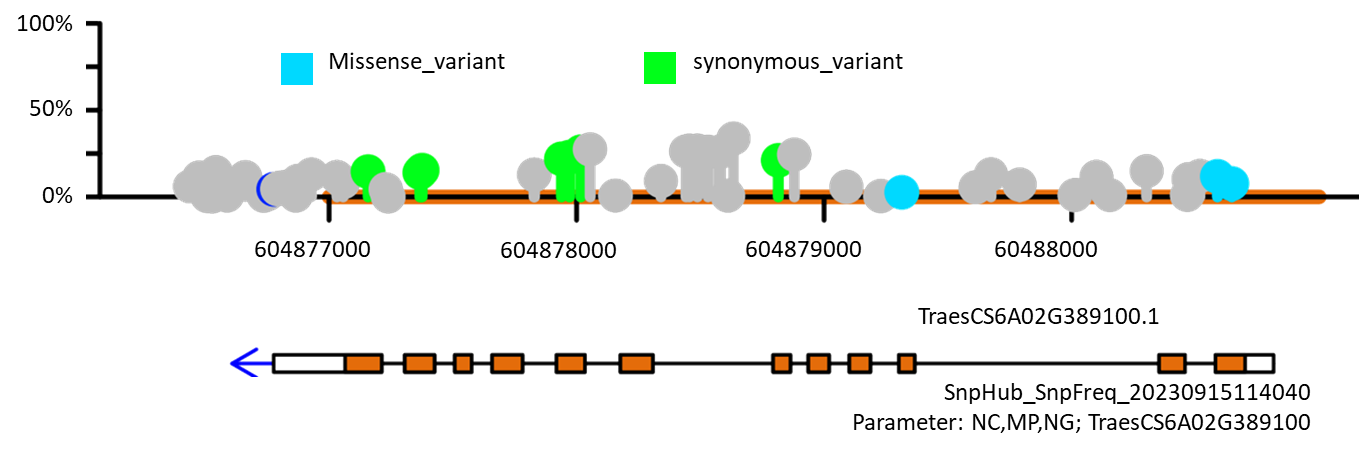
**

**Figure S5.** Schematic of *TaNDUFA9* variation in wheat union database [33].
